# Fasting-Induced Upregulation of MKP-1 Modulates the Hepatic Response to Feeding

**DOI:** 10.3390/nu13113941

**Published:** 2021-11-04

**Authors:** Jacob Sellers, Abigail Brooks, Savanie Fernando, Gabrielle Westenberger, Sadie Junkins, Shauri Smith, Kisuk Min, Ahmed Lawan

**Affiliations:** 1 Department of Biological Sciences, University of Alabama in Huntsville, Huntsville, AL 35899, USA; jas0103@uah.edu (J.S.); ajb0064@uah.edu (A.B.); ysf0001@uah.edu (S.F.); gabby.westenberger@gmail.com (G.W.); smj0024@uah.edu (S.J.); shaurismith@gmail.com (S.S.); 2 Department of Kinesiology, University of Texas at El Paso, El Paso, TX 79968, USA; kmin@utep.edu

**Keywords:** fasting, mitogen-activated protein kinase, protein tyrosine phosphatase, obesity, cholesterol, fatty acid biosynthesis, sterol regulatory element binding protein

## Abstract

The liver plays a key role in whole-body, glucose and lipid homeostasis. Nutritional signals in response to fasting and refeeding regulate hepatic lipid synthesis. It is established that activation of mitogen-activated protein kinase (MAPK) phosphatase-1 (MKP-1) in response to overnutrition regulates MAPK-dependent pathways that control lipid metabolism in the liver. However, the regulatory mechanisms and the impact of the actions of MKP-1 in hepatic response to fasting remains unclear. We investigated the effect of fasting on the expression of MKP-1 and the impact on hepatic response to feeding. In this study, we demonstrate that fasting stress induced upregulation of hepatic MKP-1 protein levels with a corresponding downregulation of p38 MAPK and JNK phosphorylation in mouse livers. We found that MKP-1-deficient livers are resistant to fasting-induced hepatic steatosis. Hepatic MKP-1 deficiency impaired fasting-induced changes in the levels of key transcription factors involved in the regulation of fatty acid and cholesterol metabolism including *Srebf2* and *Srebf1c*. Mechanistically, MKP-1 negatively regulates *Srebf2* expression by attenuating p38 MAPK pathway, suggesting its contribution to the metabolic effects of MKP-1 deficiency in the fasting liver. These findings support the hypothesis that upregulation of MKP-1 is a physiological relevant response and might be beneficial in hepatic lipid utilization during fasting in the liver. Collectively, these data unravel some of the complexity and tissue specific interaction of MKP-1 action in response to changes in nutritional cues, including fasting and excess nutrients

## 1. Introduction

Globally the incidence of overweight and obese individuals increases. It has been predicted that by the year 2030 overweight and obese individuals will constitute over half of the world’s population [1,2]. Despite extensive studies in the pathogenesis of obesity the precise causes of human obesity are unclear. Obesity predisposes to the development of insulin resistance, type 2 diabetes, non-alcoholic fatty liver disease and cardiovascular diseases [2,3]. The molecular mechanisms and the cellular events responsible for dysregulation of metabolic homeostasis, glucose and lipid metabolism resulting from signals from many peripheral tissues are nevertheless not clear. Dysregulated hepatic gluconeogenesis is an important feature of insulin resistance that contributes to the pathology of hyperglycemia and type 2 diabetes. Hepatic gluconeogenesis plays a critical role in providing glucose from non-carbohydrate precursors during prolonged fasting [4]. Fasting is an adaptive response of metabolism when there is inadequate nutrient consumption [4].

The transcription factors family sterol regulatory element binding proteins (*Srebfs*) modulate genes involved in cholesterol and fatty acid synthesis. Three *Srebf* (gene *Srebf*) isoforms have been identified; *Srebf1a*, *Srebf1c* and *Srebf2*. *Srebf2* is the major regulator of cholesterol homeostasis, while *Srebf1c* mainly regulates synthesis of fatty acids [5,6]. However, *Srebf1a* regulates both cholesterol metabolism and lipogenesis [5,6]. Transcriptionally the membrane-bound *Srebfs* are inactive. In the endoplasmic reticulum (ER), *Srebfs* form a complex by interacting with *Srebf* cleavage-activating protein (Scap) [5,6]. Under states of sterol depletion, Scap/*Srebf* complex is transported to the Golgi apparatus where the *Srebfs* are prepared by the site 1 (S1P) and site 2 (S2P) protease to release the mature forms of the proteins. These functional forms of the *Srebfs* are transferred into the nucleus, where they bind to the promoters of *Srebf* target genes to promote the transcription of genes involved in the biosynthesis of lipids [6,7]. In the liver, mitogen-activated protein kinases (MAPKs) contribute to a host of processes that regulate hepatic metabolism [3,8,9]. It has been shown that *Srebf1c* is phosphorylated by p38 MAPK and extracellular signal- regulated kinase (ERK) in HepG2 cells [10]. How MAPKs regulate *Srebfs* in the liver *in vivo* is not clear. The MAPKs are inactivated by the MAPK phosphatases (MKPs) [11]. However, how the MAPK/MKP balance is controlled in the liver under fasting and how these activities contribute to the regulation of *Srebfs* in the control of cholesterol and fatty acid synthesis remains unclear.

The plasma free fatty acids are enhanced in obesity. In rodents, fatty acid synthesis occurs in both the liver and adipose tissue [4,12]. During fasting in rodents there is decrease synthesis of fatty acids and cholesterol [4]. Fasting and refeeding a high-carbohydrate low-fat diet paradigm is used as model to study the regulation of substrate flux in the fatty acid and cholesterol synthesis pathways [13]. Fasting reduces and refeeding a high-carbohydrate low-fat enhances the activities of lipogenic genes [12]. It has been reported that refeeding a high-carbohydrate low-fat diet induces the synthesis of fatty acids to levels higher than the fed state, however, cholesterol synthesis returns to fed state levels [14]. Fasting reduces nuclear *Srebf1c* and the expression of fatty acid biosynthetic genes [15]. Circulating insulin and glucagon levels affect *Srebf1c* transcription [16]. The transcription of *Srebf2* is affected by the availability of cholesterol and expression of *Srebf1c* is affected by fatty acids [17]. Recently, Linden et al. showed that insulin stimulation of lipogenic genes is partly mediated by *Srebf1c* [18]. Similarly, Miao et al. demonstrated that *Srebf2* mediates insulin action in the synthesis of hepatic cholesterol [19].

The goal of the present study was to determine the effect of nutritional status; fed, fasting and refeeding on the expression of MKP-1 and the effect of hepatocyte-specific deletion on liver metabolism in response to fasting. Mice deficient for MKP-1 in the liver have been genetically characterized elsewhere [20].

## 2. Materials and Methods

### 2.1. Reagents and Antibodies

All reagents were purchased from standard chemical vendors. The following antibodies were used; phospho-p38 MAPK (#9215S), phospho-JNK1/2 (#4668S), p38 MAPK (#9228s), JNK (#3708s), beta-actin (#8457s), α-tubulin (#2125s), Histone H3 (#4499s), were obtained from Cell Signaling Technology. Antibodies to MKP-1 (#sc-373841) were obtained from Santa Cruz Biotechnology. Antibodies to *Srebf2* (#ab-30682) were obtained from ABCAM. Antibodies to SREBP1 (#BD-557036) were obtained from BD Bioscience.

### 2.2. Animal Studies

The University of Alabama in Huntsville Institutional Animal Care and Use Committee approved all animal studies. We published the generation and genetic characterization of MKP-1 liver-specific knockout (MKP1-LKO) mice [20]. These mice were kindly provided by Dr. Anton Bennett, Yale School of Medicine. The fasting and refeeding experiments comprised of five to twelve male MKP1-LKO and *Mkp-1^fl/fl^* mice in each treatment group. The treatment groups comprised of mice fed *ad libitum* with rodent lab diet (Lab supply, Nothlake, TX, USA) or fasted for 24 h prior to sacrifice. For the refeeding experiments, MKP1-LKO and *Mkp-1^fl/fl^* mice were fasted for 24 h and refed a either a chow (custom low fat purified rodent diet) or high carbohydrate/low fat diet (AIN-93M, Dyets, Bethlehem, PA, USA) for 3 h *ad libitum* prior to sacrifice. We used seven to eight weeks old male mice for all the fasting and refeeding experiments. MKP1-LKO and *Mkp-1^fl/fl^* mice were fasted at zeitgeber time (ZT) 8 to (ZT) 8 and then sacrificed between (ZT) 8 and (ZT) 10. MKP1-LKO and *Mkp-1^fl/fl^* mice were fasted (ZT) 8 and refed at (ZT) 8 to (ZT) 11 then sacrificed at (ZT) 11 to (ZT) 13. Randomly fed MKP1-LKO and *Mkp-1^fl/fl^* mice were sacrificed between (ZT) 8 and (ZT) 10. All protein and RNA isolations from liver samples were performed between (ZT) 0 and (ZT) 9.

We chose the high carbohydrate low fat diet (Table 1) in this study because substrate flux through the fatty acid and cholesterol biosynthesis pathway are significantly decreased by fasting, hence refeeding a high carbohydrate low fat diet stimulates fatty acid and cholesterol synthesis. Chow diet was used as control (Table 2).

### 2.3. Cell Culture, Nuclear-Cytoplasmic Isolation and Transfections

HEK 293 cells were cultured at 37 °C with 5% CO_2._ HEK 293 were co-transfected with *Srebf2* and either MKK6 (WT) (Addgene, #13517), MKK7 (WT) (Addgene, #14538), or constitutively active mutants of the upstream activators of p38 MAPK, MKK6 (EE) (Addgene, #13518), was generated by the substitution of Ser-207 and Thr-211 with glutamic acid; JNK, MKK7 (EE) (Addgene, # 14540), was generated by the substitution of Ser-198 and Thr-202 by glutamic acid or dominant negative mutants of p38 MAPK, MKK6 (AA) (Addgene, #13519), was generated by the substitution of Ser-207 and Thr-211 with alanine; JNK, MKK7 (AA) (Addgene, #14539), was generated by the substitution of Ser-198 and Thr-202 by alanine [21]. MKK6 selectively enhance p38 MAPK activity while MKK7 selectively increase JNK activity. Plasmids were transfected with Lipofectamine 3000 (Invitrogen, Carlsbad, CA, USA) according to the manufacturer’s instructions. Nuclear and cytoplasmic fractions was isolated using the NE-PER Nuclear and Cytoplasmic Extraction Reagents kit (Thermofisher Scientific, San Francisco Bay Area, San Francisco, CA, USA) according to the manufacturer’s protocol.

### 2.4. RNA Extraction and Real-Time Quantitative PCR Analysis

RNA was isolated from liver tissue from male *Mkp-1^fl/fl^* and MKP1-LKO mice using an RNeasy kit (Qiagen, Valencia, CA, USA, according to the manufacturer’s instructions. A total of 1 μg RNA was reverse transcribed to generate cDNA using a reverse transcriptase PCR kit (Applied Biosystems, Foster City, CA, USA. Real-time quantitative PCR was performed in triplicate with the Applied Biosystems 7500 Fast RT-PCR system and, TaqMan and SYBR Green gene expression master mix with the following primer pairs: *Srebf2*, 5′-GCAGCAACGGGACCATTCT-3′ and 3′-CCCCATGACTAAGTCCTTCAACT-5′; 18S, 5′-ACCGCAGCTAGGAATAATGGA-3′ and 3′-GCCTCAGTTCCGAAAACCA-5′; *Srebf1c*, 5′-ATCTCCTAGAGCGAGCGTTG and 3′-TATTTAGCAACTGCAGATATCCAAG; HMGCR, 5′-CTTTCTAGAGCGAGTGCATTAGCAAAGTTTG and 3′-GCGTCAAGAGTGAATGTGGGGCCCAGGATTG; HMGCS, 5′-CCTACCGCAAGAAGATCCAG-3′ and 3′-GAAAGGCTGGTTGTTTCCAG-5′; Ldlr, 5′-GAAAATGACTCAGACGAACAAGGCTG and 3′-TCATCAGAGCCATCTAGGCAATCTC; CPT1α, 5′-TGTCAAAGATACCGTGAGCAG-3’ and 5′-GCCCACCAGGATTTTAGCTT-3’; PCK1, 5′-CTAACTTGGCCATGATGAACC-3′ and 3′-CTTCACTGAGGTGCCAGGAG-5′; CCL2, 5′-TTAAAAACCTGGATCGGAACCAA-3′ and 3′-GCATTAGCTTCAGATTTACGGGT-5′; LXRα, 5′-GGATAGGGTTGGAGTCAGCA-3′ and 3′-CTTGCCGCTTCAGTTTCTTC-5′.

Cyp4a14, 5′-TTTAGCCCTACAAGGTACTTGGA-3′ and 3′-GCAGCCACTGCCTTCGTAA-5′; CD36, 5′-ATGGGCTGTGATCGGAACTG-3′ and 3′-TTTGCCACGTCATCTGGGTTT-5′; ApoB; 5′-CGTGGGCTCCAGCATTCTA-3′ and 3′-TCACCAGTCATTTCTGCCTTTG-5′; ApoE, 5′-CTGACAGGATGCCTAGCCG-3′ and 3′-CGCAGGTAATCCCAGAAGC-5′; Cyp4a10, 5′-TTCCCTGATGGACGCTCTTTA-3′ and 3′-GCAAACCTGGAAGGGTCAAAC-5′. All relative gene expression levels were analyzed using the ∆Ct method and normalized to 18S. TaqMan primers and gene expression master mix from Applied Biosystems were used for FASN and quantitation.

### 2.5. Measurement of Fasting Blood Glucose and Hepatic Lipids

Male chow fed *Mkp-1^fl/fl^* and MKP1-LKO mice aged between 7 to 8 weeks old were used for the measurement of fasting plasma glucose concentrations by a glucometer (CareTouch Blood Glucose Monitoring System, Brooklyn, NY, USA). Hepatic triglycerides were determined using a triglyceride colorimetric assay kit (TG, Cayman Chemical, Ann Arbor, MI, USA) according to the manufacturer’s protocol.

### 2.6. Histological Analysis of Tissue Sections

Livers from male chow-fed and fasted male *Mkp-1^fl/fl^* and MKP1-LKO mice were isolated and then fixed in 4% paraformaldehyde in PBS and processed for paraffin sections and stained with hematoxylin and eosin.

### 2.7. Immunoblotting

Liver tissue was homogenized in RIPA buffer (25 mM Tris. HCl pH 7.4, 150 mM NaCl, 5 mM EDTA, 1% NP-40, 0.1% SDS, 1.0% sodium deoxycholic acid), supplemented with protease and phosphatase inhibitors (5 μg/mL leupeptin, 5 μg/mL aprotinin, 1 μg/mL pepstatin A, 1 mM PMSF, 1 mM benzamidine, 1 mM Na_3_VO_3_, and 10 mM NaF). Homogenates were lysed for 30 min on the shaker at 4 °C prior to clarification at 20,800 g for 30 min at 4 °C. Protein concentrations were determined by Pierce BCA Protein Assay kit (Pierce, Rockford, IL, USA). Lysates were resolved by SDS-PAGE and transferred to nitrocellulose membranes, which were incubated with phospho-specific antibodies followed by enhanced chemiluminescence or fluorescent detection.

## 3. Statistical Analysis

All data represent the mean ± SEM. Differences between groups were assessed using a student’s *t*-test or analysis of variance (ANOVA) with Bonferroni’s post-test for multiple comparisons using GraphPad Prism 9 statistical software.

## 4. Results

### 4.1. Fasting Induces MKP-1 Expression in the Liver

**Figure 1:**
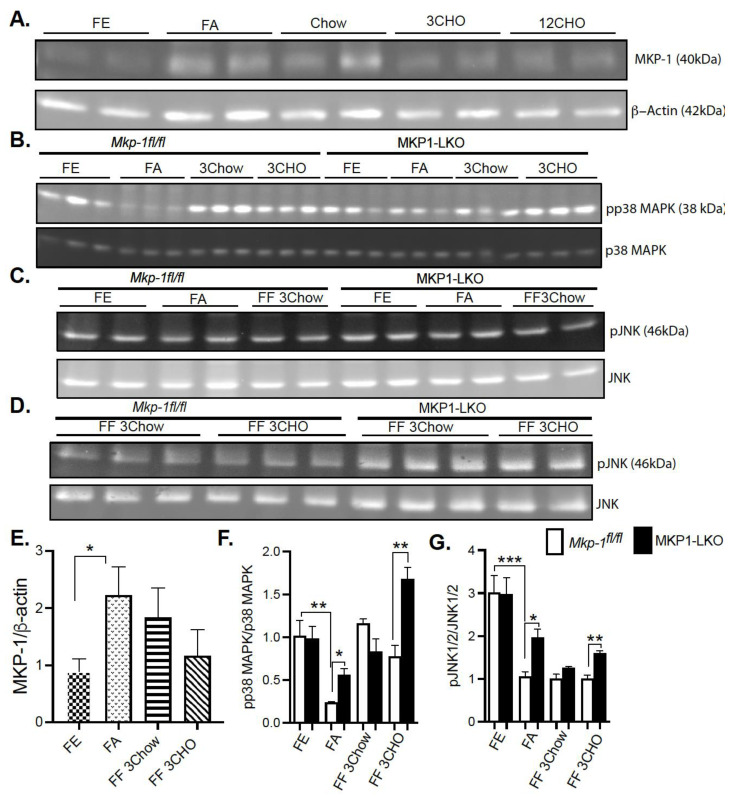
Fasting Induced MKP-1 Expression in the Livers of Mice. Liver lysates from fasted and chow and high carbohydrate/low fat diet fed *Mkp-1fl/fl* and MKP1-LKO mice were analyzed by immunoblotting (**A**,**E**) MKP-1, (**B**,**F**) pp38 MAPK, pJNK1/2 (**C**,**D**,**G**). Representative immunoblots were quantitated by densitometry for the levels of MKP-1/β-actin, phospho-p38 MAPK/p38 MAPK, phospho-JNK1/2/JNK1/2. Results represent *n* = 5–10 per genotype and data shown are the mean ± SEM; *; *p* < 0.05, **; *p* < 0.01, ***; *p* < 0.0001 as determined by analysis of variance (ANOVA) with Bonferroni’s post-test for multiple comparisons. FE; Fed, FA; Fasted FF; Refed. Open. bars, *Mkp-1fl/fl* mice; closed bars, MKP1-LKO mice.

In order to determine whether MKP-1 is metabolically regulated due to fasting-induced changes in expression, we assessed MKP-1 protein levels in the livers of mice using the fasting-refeeding paradigm. We fasted *Mkp-1^fl/fl^* mice for 24 h and refed a chow or high carbohydrate/low fat diet for 3 h. Liver tissues were assayed by immunoblotting for MKP-1 protein expression. Under fed conditions the protein expression of MKP-1 is very low (Figure 1A,E), however, hepatic MKP-1 protein levels dramatically increased >2 fold after a 24 h fast (Figure 1A,E). Refeeding for 3 h with chow diet after the 24 h fast increased hepatic MKP-1 protein levels above the fed state levels, ~1.5 fold (Figure 1A,E). After the 24 h fast, refeeding with high carbohydrate/low fat diet for 3 h increased hepatic MKP-1 protein levels above non-fasted levels, however, this was not statistically significant (Figure 1A,E). These results demonstrate that MKP-1 is induced during fasting in the livers of wild type mice.

### 4.2. Fasting-Induced Downregulation of p38 MAPK and JNK in the Liver

Since fasting induced MKP-1 expression in the livers of mice, next was to understand the effect of metabolic response to fasting on activation of stress-responsive MAPKs, p38 MAPK and JNK. We fasted MKP1-LKO mice and *Mkp-1^fl/fl^* mice for 24 h and refed a either a chow or high carbohydrate/low fat diet for 3 h. Since no apparent differences in ERK1/2 activities were found in the genetic characterization of MKP1-LKO mice [20], we focused on p38 MAPK and JNK in this study. In *Mkp-1^fl/fl^* mice the increased levels of MKP-1 during fasting was accompanied by a concomitant decrease in the phosphorylation of p38 MAPK and JNK (Figure 1B–D,F,G). In the fed state, we found comparable levels of hepatic phosphorylation of p38 MAPK and JNK in MKP1-LKO mice compared with *Mkp-1^fl/fl^* mice (Figure 1B–D,F,G). Hepatic phosphorylation of p38 MAPK and JNK decreased in both *Mkp-1^fl/fl^* and MKP1-LKO mice after 24 h fast compared with non-fasted levels (Figure 1B–D,F,G). However, MKP1-LKO mice exhibited significantly enhanced hepatic phosphorylation of p38 MAPK and JNK compared with *Mkp-1^fl/fl^* mice in the fasted state (Figure 1B–D,F,G). Refeeding a chow diet (3 h) after the 24 h fast returned hepatic phosphorylation of p38 MAPK in *Mkp-1^fl/fl^* and MKP1-LKO mice to levels comparable to the non-fasted levels (Figure 1B,F), however, JNK phosphorylation did not return to levels comparable to non-fasted levels (Figure 1C,D,G). Furthermore, there was no difference in p38 MAPK and JNK phosphorylation between the two genotypes after refeeding with chow diet (3 h) (Figure 1B–D,F,G). Interestingly, after 24 h fast, refeeding a high carbohydrate/low fat diet for 3 h significantly increased hepatic phosphorylation of p38 MAPK above non-fasted levels, and MKP1-LKO mice exhibited significantly enhanced hepatic phosphorylation of p38 MAPK compared with *Mkp-1^fl/fl^* mice (Figure 1B,F). Refeeding a high carbohydrate/low fat diet for 3 h significantly increased hepatic phosphorylation of JNK in MKP1-LKO mice compared with *Mkp-1^fl/fl^* mice, although these were below fed levels (Figure 1C,D,G). These results demonstrate that in *Mkp-1^fl/fl^* mice, fasting induced downregulation of hepatic p38 MAPK and JNK phosphorylation that is consistent with increased hepatic MKP-1 expression. This is the first study that showed enhanced MKP-1 expression and MAPK regulation by fasting-induced stress in livers of mice.

### 4.3. Physiologic Parameters of MKP-1-Deficient Liver after Fasting and Refeeding

**Figure 2:**
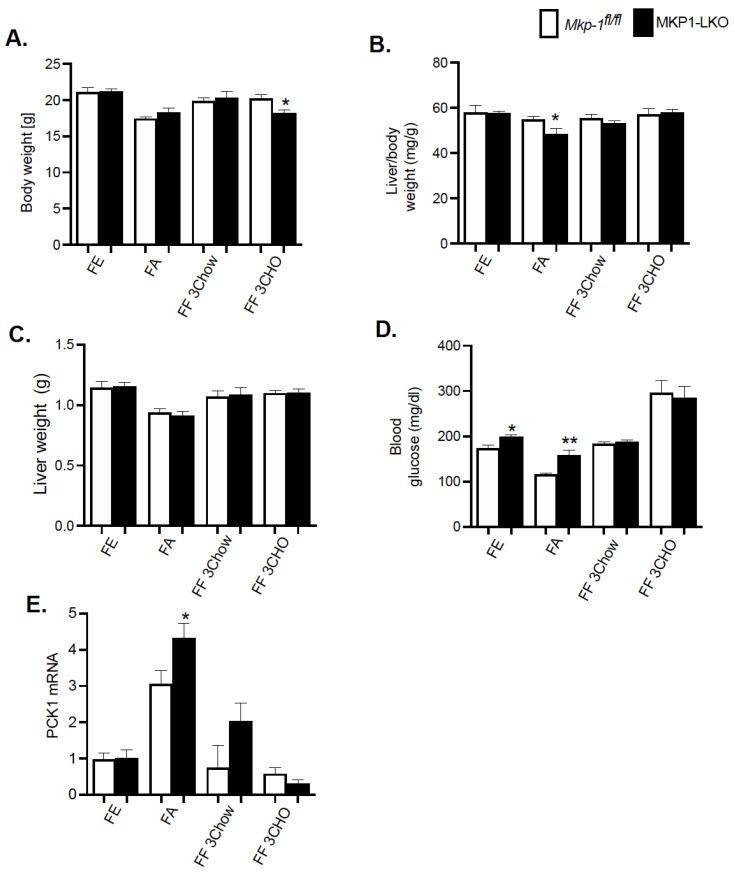
Physiological Parameters of MKP1-LKO mice after Fasting and Refeeding. (**A**) Body weight of fasted, chow- or high carbohydrate/low fat diet fed male *Mkp-1fl/fl* and MKP1-LKO mice (**B**) Liver to body weight ratio and (**C**) Liver weight (**D**) Fasting blood glucose (**E**) mRNA expression of hepatic PCK1. *Mkp-1fl/fl* and MKP1-LKO mice (*n* = 5–8 per genotype). Data are represented as mean ± SEM; *; *p* < 0.05, **; *p* < 0.01 as determined by analysis of variance (ANOVA) with Bonferroni’s post-test for multiple comparisons. FE: Fed, FA: Fasted FF: Refed. Open bars, *Mkp-1fl/fl* mice; closed bars, MKP1-LKO mice.

We fasted MKP1-LKO mice and *Mkp-1^fl/fl^* mice for 24 h and refed a either a chow or high carbohydrate/low fat diet for 3 h to establish the impact of hepatic MKP-1 deficiency on the metabolic response to fasting. MKP1-LKO mice showed comparable body weight in the fed, fasted, or refed for 3 h chow or high carbohydrate/low fat diet to those of *Mkp-1^fl/fl^* mice (Figure 2A). Surprisingly, when refed a high carbohydrate/low fat diet for 3 h, MKP1-LKO mice showed small but significant reduction in body weight compared with *Mkp-1^fl/fl^* mice (Figure 2A). Also, MKP1-LKO mice displayed comparable liver weight when fed, or refed chow or high carbohydrate/low fat diet to those of *Mkp-1^fl/fl^* mice. However, in the fasted state, MKP1-LKO mice exhibited small but significant decrease in liver weight compared with *Mkp-1^fl/fl^* mice (Figure 2C). MKP1-LKO mice exhibited comparable blood glucose levels in the fed, or refed chow or high carbohydrate/low fat diet to those of *Mkp-1^fl/fl^* mice (Figure 2D). Consistent with our previous findings, MKP1-LKO mice exhibited significant fasting hyperglycemia compared with *Mkp-1^fl/fl^* mice (Figure 2D). We further examined the mRNA expression of the rate-limiting enzyme involved in hepatic glucose production, phosphoenolpyruvate carboxykinase (*Pck1*) in the liver. In the fasted state, we found that *Pck1* was significantly enhanced in MKP1-LKO mice compared with *Mkp-1^fl/fl^* mice (Figure 2E). These findings suggest that MKP1-LKO mice are hyperglycemic.

### 4.4. Protection from Fasting-Induced Hepatic Steatosis in MKP-1-Deficient Liver

**Figure 3:**
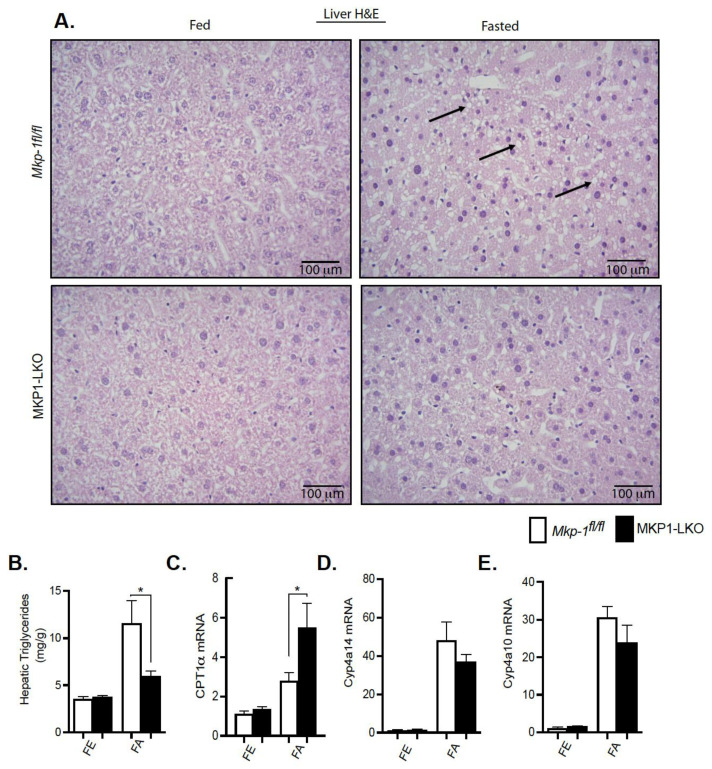
Protection from fasting-induced hepatic steatosis in MKP1-LKO mice. Representative hematoxylin and eosin staining of liver sections of chow-fed (left panel) and fasted (right panel) *Mkp-1fl/fl* and MKP1-LKO mice (**A**) (*n* = 5 per genotype). Arrows show lipid droplets. (400× Magnification). Hepatic triglycerides (TG) from chow-fed and fasted *Mkp-1fl/fl* and MKP1-LKO mice (**B**) (*n* = 5 per genotype). mRNA expression of hepatic CPT1α (**C**), Cyp4a14, (**D**) and Cyp4a10 (**E**) from fed and fasted *Mkp-1fl/fl* and MKP1-LKO mice (**C**) (*n* = 6 per genotype). Data shown are the mean ± SEM; *; *p* < 0.05, as determined by student t test. FE: Fed, FA: Fasted FF: Refed. Open bars, *Mkp-1fl/fl* mice; closed bars, MKP1-LKO mice.

We showed that MKP-1 is upregulated during fasting in the liver. Since fatty acids carried to the liver serve as an alternate source of energy during fasting, we examined the role of MKP-1 in fasting-induced hepatic steatosis. We fasted MKP1-LKO mice and *Mkp-1^fl/fl^* mice for 24 h. In the fed state, the livers of MKP1-LKO and *Mkp-1^fl/fl^* mice are comparable (Figure 3A, left panel). Interestingly, after 24 h fast, the livers of MKP1-LKO mice exhibited protection from the development of hepatic steatosis compared with *Mkp-1^fl/fl^* mice as assessed by hematoxylin and eosin staining (Figure 3A, right panel). This is consistent with small but significant reduction in liver weight in MKP1-LKO mice (Figure 2B). Consistent with resistance to hepatic steatosis MKP1-LKO mice exhibited a significant reduction in hepatic triglycerides (TGs) (Figure 3B) compared with *Mkp-1^fl/fl^* mice. Furthermore, analysis of a key rate-limiting enzyme in the hepatic fatty acid β-oxidation pathway showed that fasted MKP1-LKO mice exhibited enhanced hepatic expression levels of carnitine palmitoyltransferase (CPT1α) compared with *Mkp-1^fl/fl^* mice (Figure 3C). Furthermore, we determined the expression levels of oxidative genes; cytochrome P450 omega-hydroxylase 4a14 (Cyp4a14) and 4a10 (cyp4a10) in the livers of MKP1-LKO mice. In the fed state, no differences were observed in the expression of Cyp4a14 and Cyp4a10 in the livers of MKP1-LKO and *Mkp-1^fl/fl^* mice (Figure 3D,E). However, in the fasted state, the hepatic expression of Cyp4a14 and Cyp4a10 dramatically increased (~30 to 40-fold) in both of MKP1-LKO and *Mkp-1^fl/fl^* mice (Figure 3D,E), consistent with previous reports [22]. However, no significant differences between the two genotypes (Figure 3D,E). These results suggest that fasting-induced upregulation of MKP-1 modulates hepatic fatty acid catabolism thereby promoting accumulation of fat in the liver. This is consistent with our previous findings where overexpression of MKP-1 in obesity negatively regulates hepatic triglyceride metabolism [3].

### 4.5. Regulation of Srebf2 and Its Target Genes during Fasting in MKP-1-Deficient Liver

**Figure 4:**
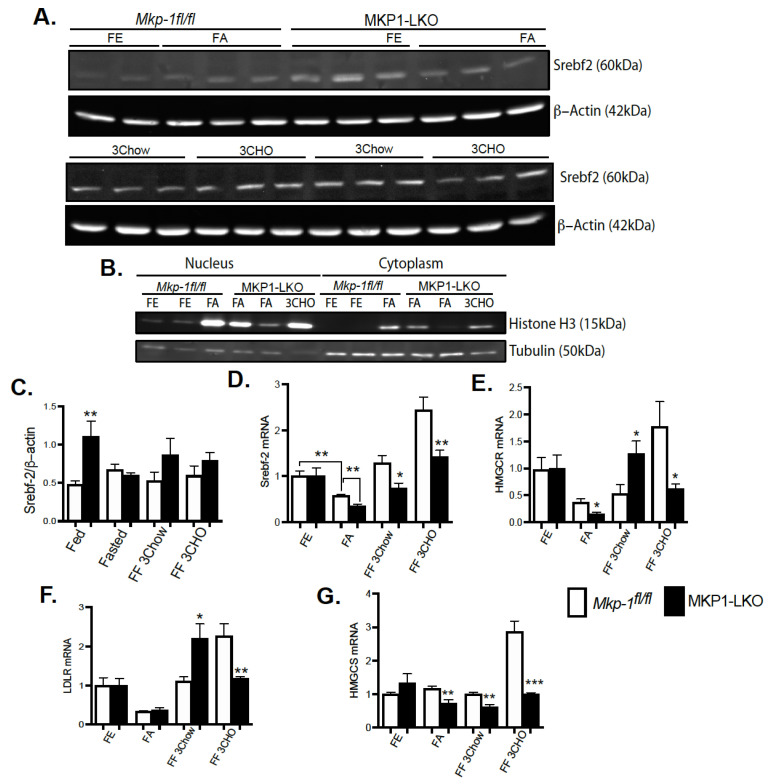
*Srebf-2* Expression and its Target Genes in Fasted MKP1-LKO Livers. (**A**) Nuclear liver extract from fasted and chow- and high carbohydrate/low fat diet refed *Mkp-1fl/fl* and MKP1-LKO mice were analyzed by immunoblotting. Immunoblots were quantitated by densitometry for the levels of *Srebf-2*/β-actin, (**A**,**C**). (**B**) Immunoblots analysis of nuclear and cytoplasmic fraction from liver tissue. mRNA expression of hepatic lipid regulatory genes from fasted and chow- and high carbohydrate/low fat diet refed *Mkp-1fl/fl* and MKP1-LKO mice, *Srebf-2* (**D**) HMGCR (**E**), LDLR (**F**) and HMGCS (**G**). Results represent *n* = 6 per genotype and data shown are the mean ± SEM; *; *p* < 0.05, **; *p* < 0.01, *** *p* < 0.0001 as determined by analysis of variance (ANOVA) with Bonferroni’s post-test for multiple comparisons. FE: Fed, FA: Fasted FF: Refed. Open bars, *Mkp-1fl/fl* mice; closed bars, MKP1-LKO mice.

To establish the impact of hepatic MKP-1 deficiency on the metabolic response to fasting, we fasted MKP1-LKO mice and *Mkp-1^fl/fl^* mice for 24 h and refed either a chow or high carbohydrate/low fat diet for 3 h. In the MKP1-LKO livers, nuclear proteins levels of *Srebf2* were similar to *Mkp-1^fl/fl^* mice after 24 h of fasting. The expression of Histone H3 and Tubulin demonstrate successful isolation of extracts from nucleus and cytoplasm (Figure 4B). Refeeding either a chow or high carbohydrate/low fat diet for 3 h caused a small increase in *Srebf2* levels in MKP1-LKO livers (Figure 4A,C), although not statistically significant compared with *Mkp-1^fl/fl^* mice. In MKP1-LKO livers, nuclear *Srebf2* protein levels were significantly increased in the nonfasted state compared with *Mkp-1^fl/fl^* mice (Figure 4A,C). At the mRNA level, hepatic *Srebf2* level significantly decreased after a 24 h fast in *Mkp-1^fl/fl^* mice compared with nonfasted state (Figure 4D), this was further diminished in MKP1-LKO mice (Figure 4D). When mice were refed either a chow or high carbohydrate/low fat diet for 3 h, in *Mkp-1^fl/fl^* mice, the hepatic expression of *Srebf2* increased above nonfasted levels (Figure 4D). In the livers of MKP1-LKO mice, *Srebf2* significantly decreased compared with *Mkp-1^fl/fl^* livers under both refed conditions (Figure 4D). The mRNA levels of HMGCR and LDLR were reduced ~2-fold after a 24 h fast in the livers of MKP1-LKO and *Mkp-1^fl/fl^* mice (Figure 4D,E), consistent with previous reports [19]. When mice were refed a chow diet for 3 h, in *Mkp-1^fl/fl^* mice, the expression of these genes increased near nonfasted levels (Figure 4D,E). In the livers of MKP1-LKO mice, HMGCR and LDLR significantly increased above nonfasted compared with *Mkp-1^fl/fl^* livers (Figure 4D,E). Interestingly, when mice were refed a high carbohydrate/low fat diet for 3 h, in *Mkp-1^fl/fl^* livers, levels of HMGCR and LDLR increased above nonfasted levels by ~ 2.5-fold (Figure 4D,E) and this effect was blunted in the livers of MKP1-LKO mice (Figure 4D,E). Following 24-fast, the gene expression levels of hepatic HMGCS were significantly reduced in *Mkp-1^fl/fl^* mice, however, this was further diminished in MKP1-LKO mice (Figure 4F). When mice were refed a chow diet for 3 h, the mRNA levels of hepatic HMGCS increased to non-fasted levels in *Mkp-1^fl/fl^* mice, (Figure 4F). However, the mRNA levels of HMGCS significantly decreased in MKP1-LKO mice (Figure 4F). When mice were refed a high carbohydrate/low fat diet, the hepatic HMGCR and HMGCS mRNA levels significantly reduced (3 h refed) in MKP1-LKO compared with *Mkp-1^fl/fl^* mice (Figure 4D,F). These results suggests that hepatic MKP-1 negatively regulates *Srebf2* expression and demonstrates that hepatic MKP-1 deficiency impairs fasting-induced changes in the levels of key transcription factors involved in the regulation of cholesterol metabolism.

### 4.6. Reduced Expression of Hepatic Lipogenic Genes in Fasted and Refed MKP-1-Defcient Liver

**Figure 5:**
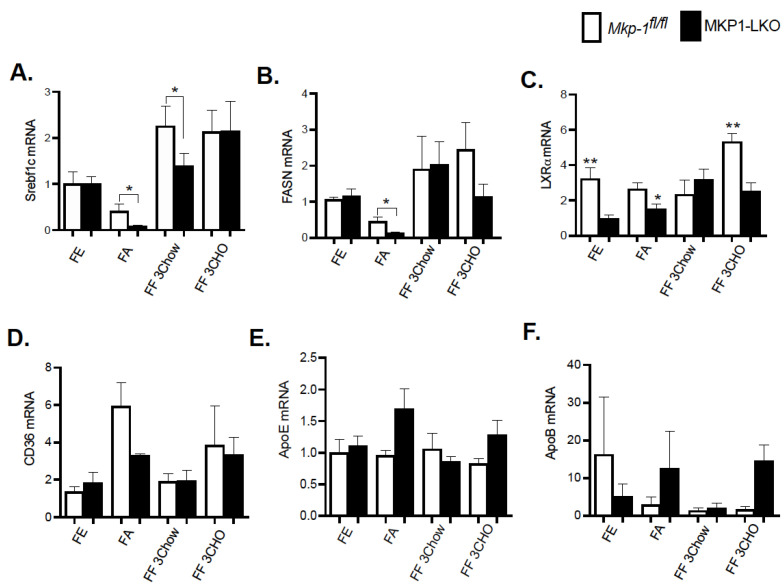
Reduced Expression of Hepatic Lipogenic Genes in Fasted and Refed MKP1-LKO Livers. mRNA expression of hepatic lipogenic and fatty acid genes from fasted and chow- and high carbohydrate/low fat diet refed *Mkp-1fl/fl* and MKP1-LKO mice, *Srebf1c* (**A**), FASN (**B**), LXRα (**C**), CD36 (**D**), ApoE (**E**) and ApoB (**F**). Results represent *n* = 6 per genotype and data shown are the mean ± SEM; *; *p* < 0.05, **, *p* < 0.01, as determined by analysis of variance (ANOVA) with Bonferroni’s post-test for multiple comparisons. FE: Fed, FA: Fasted FF: Refed. Open bars, *Mkp-1fl/fl* mice; closed bars, MKP1-LKO mice.

In the liver, lipid and energy metabolism is mediated by many transcription factors. We assessed the gene expression of key hepatic lipid regulatory enzymes to determine the mechanisms by which hepatic MKP-1 deficiency impacts the metabolic fate of fatty acids in response to fasting-induced changes. The de novo hepatic synthesis of fatty acids and triacylglycerols is regulated by *Srebf1c* by inducing the expression of lipogenic genes. We assessed the mRNA levels of *Srebf1c*, LXRα and fatty acid synthase (FASN), which is under the control of *Srebf1c*. MKP1-LKO mice and *Mkp-1^fl/fl^* mice were fasted for 24 h and refed a either a chow or high carbohydrate/low fat diet for 3-h to determine the impact of hepatic MKP-1 deficiency on the expression of *Srebf1c* and its target genes. In the fed state, quantitative PCR analysis of hepatic *Srebf1c* mRNA expression showed comparable levels of *Srebf1c* in MKP1-LKO and *Mkp-1^fl/fl^* mice (Figure 5A). As anticipated, the hepatic expression of *Srebf1c* decreased in *Mkp-1^fl/fl^* mice following 24-h fast, however, the expression levels were significantly diminished in the livers of MKP1-LKO mice (Figure 5A). Consistent with previous reports [18], refeeding either a chow or high carbohydrate/low fat diet for 3 h caused ~2.5-fold increase in hepatic *Srebf1c* levels in *Mkp-1^fl/fl^* mice (Figure 5A), however, this was impaired (chow refed) in MKP1-LKO mice (Figure 5A). Consistent with the levels of *Srebf1c* in the fed state, the livers of MKP1-LKO and *Mkp-1^fl/fl^* mice displayed comparable mRNA levels of FASN (Figure 5B). After 24-fast, the gene expression levels of hepatic FASN significantly reduced in *Mkp-1^fl/fl^* mice, however, the levels further decreased in MKP1-LKO mice (Figure 5B). When mice were refed a chow diet for 3 h, the mRNA levels of hepatic FASN increased above non-fasted levels in MKP1-LKO and *Mkp-1^fl/fl^* mice, (Figure 5B). However, the hepatic mRNA levels of FASN decreased in MKP1-LKO mice when mice were refed a high carbohydrate/low fat diet for 3 h, compared with *Mkp-1^fl/fl^* mice (Figure 5B). To further examine the effect of hepatic MKP-1 deficiency on lipogenesis we assessed the hepatic mRNA expression of LXRα. Under both fed, fasted and refed conditions, the levels of hepatic LXRα significantly reduced in MKP1-LKO mice compared with *Mkp-1^fl/fl^* mice (Figure 5C). In addition, the mRNA expression of hepatic CD36, a gene involved in fatty acid absorption showed no difference between MKP1-LKO mice and *Mkp-1^fl/fl^* mice (Figure 5D). Considering that fasted MKP1-LKO exhibit reduced triglyceride accumulation, we assessed the expression of genes involved in very-low density lipoprotein (VLDL) secretion. No difference was observed in the mRNA expression of ApoE and ApoB in the livers of MKP1-LKO mice and *Mkp-1^fl/fl^* mice (Figure 5E,F). These results suggest that hepatic MKP-1 deficiency impairs fasting-induced changes in the levels of key transcription factors involved in the regulation of fatty acid synthesis and hepatic lipogenesis.

### 4.7. Enhanced Hepatic Inflammatory Response after Fasting and Refeeding a High CHO Diet in MKP-1-Deficient Liver

**Figure 6:**
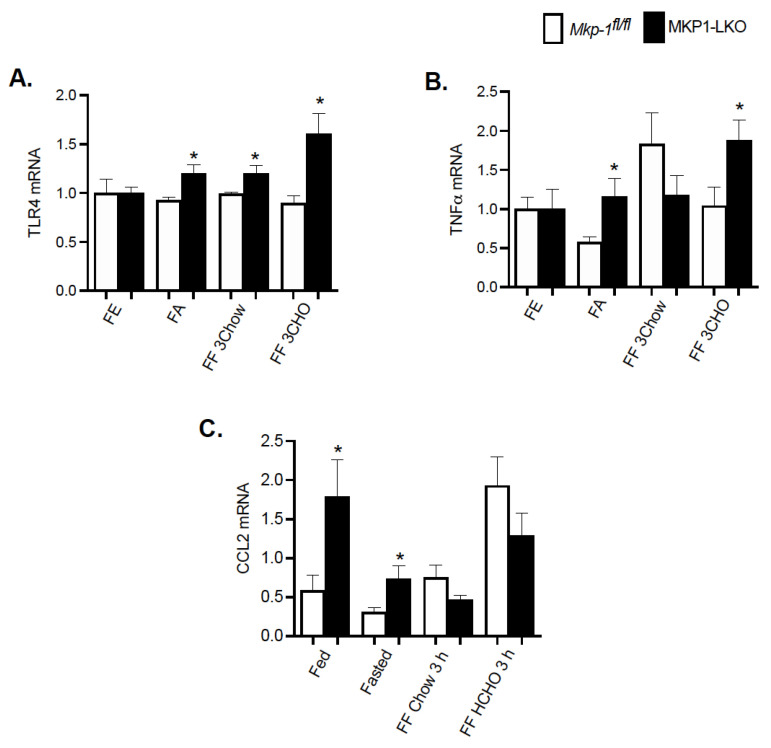
Enhanced Hepatic Inflammatory Response after Refeeding a High CHO Diet in MKP1-LKO Mice. mRNA expression of hepatic inflammatory genes from fasted and chow- and high carbohydrate/low fat diet refed *Mkp-1fl/fl* and MKP1-LKO mice. TLR4 (**A**), TNFα (**B**), and CCL2 (**C**). Results represent *n* = 6 per genotype and data shown are the mean ± SEM; *; *p* < 0.05, as determined by analysis of variance (ANOVA) with Bonferroni’s post-test for multiple comparisons. FE: Fed, FA: Fasted, FF: Refed. Open bars, *Mkp-1fl/fl* mice; closed bars, MKP1-LKO mice.

To understand the effect of fasting-refeeding paradigm on the hepatic inflammatory response in MKP1-LKO mice, we examined the gene expression levels of key inflammatory markers. It is known that MKP-1 is a negative regulator of inflammation [11]. MKP1-LKO mice and *Mkp-1^fl/fl^* mice were fasted for 24 h and refed a either a chow or high carbohydrate/low fat diet for 3 h. In the fed state, quantitative PCR analysis of hepatic TLR4 and TNFα mRNA expression showed comparable levels of TLR4 and TNFα in MKP1-LKO and *Mkp-1^fl/fl^* mice (Figure 6A,B). However, in the fed state, the mRNA expression of C-C chemokine ligand (CCL2, also known as monocyte chemoattractant protein 1 (MCP1) was significantly enhanced in MKP1-LKO compared with *Mkp-1^fl/fl^* mice (Figure 6C). After 24 h-fast, the gene expression levels of hepatic TLR4, TNFα and CCL2 significantly increased in MKP1-LKO compared with *Mkp-1^fl/fl^* mice (Figure 6A–C). When mice were refed a chow diet for 3 h, the mRNA levels of hepatic TLR4 significantly increased in MKP1-LKO mice compared with *Mkp-1^fl/fl^* mice, (Figure 6A), although the mRNA levels of TNFα and CCL2 were reduced in the MKP1-LKO mice (Figure 6B,C). When refed a high carbohydrate/low fat diet for 3 h, the gene expression levels of hepatic TLR4 and TNFα significantly increased in MKP1-LKO compared with in *Mkp-1^fl/fl^* mice (Figure 6A,B), whereas that of CCL2 reduced in MKP1-LKO mice. These results demonstrate that MKP-1 negatively regulates hepatic TLR4 and TNFα expression in response to refeeding a high carbohydrate/low fat diet.

### 4.8. p38 MAPK Mediates MKP-1 Regulation of Srebf2

**Figure 7:**
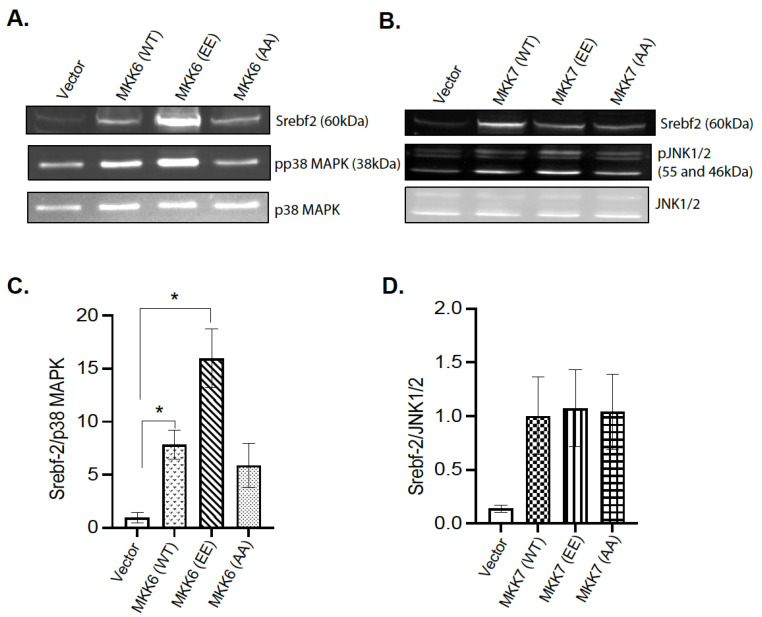
p38 MAPK Mediates MKP-1 Regulation of *Srebf-2*. 293 cells were cotransfected with vector or wild type SREBP-2 with constitutively active mutants of MKK6 and MMK7. 293 cell lysates were immunoblotted for phospho-(p)p38 MAPK (**A**,**C**), pJNK1/2 (**B**,**D**) and *Srebf-2*. Results represent *n* = 3–4 independent experiments and data shown are the mean ± SEM; *; *p* < 0.05; as determined by analysis of variance (ANOVA) with Bonferroni’s post-test for multiple comparisons.

The observation that hepatic MKP-1 negatively regulates *Srebf2* expression led us to examine the mechanism of MAPK dependency. Initial transfection experiments using the AML12 mouse hepatocyte cell line were not successful. To determine the p38MAPK and JNK dependency for the changes in *Srebf2* expression, 293 cells were transiently cotransfected with wild type *Srebf2* and activating mutants of MKK6 and MKK7 in order to constitutively activate p38 MAPK and JNK respectively (Figure 7A–D). Activating mutants of MKK6 enhanced *Srebf2* protein expression (Figure 7A,C). In contrast, we did not observe any significant change in the protein expression of *Srebf2* with an MKK7-activating mutant (Figure 7B,D). These results demonstrate that MKP-1 negatively regulates *Srebf2* expression by opposing p38 MAPK pathway.

## 5. Discussion

In this study, we demonstrate that MKP-1 protein expression is increased in its levels of protein expression in livers of mice after fasting and MKP-1 deficiency alters the hepatic response to fasting. Interestingly, in fasted human subjects, MKP-1 has been shown to be enhanced in peripheral blood mononuclear cells [23]. MKP-1 plays a major role in glucose and lipid metabolism, and in models of diet-induced obesity MKP-1 is upregulated. However, the regulatory mechanisms of MKP-1 expression in many tissues remains unclear. These findings demonstrate that MKP-1 protein expression is surprisingly regulated by both fasting and feeding signals. Consistent with increased MKP-1 protein expression, hepatic p38 MAPK and JNK phosphorylation were downregulated after fasting. This is the first study that demonstrated the effect of fasting on MKP-1 expression and p38 MAPK and JNK regulation in mouse liver. However, one study showed enhanced ERK phosphorylation in the hypothalamic arcuate nucleus of fasted wild type mice [24]. Similarly, in fasted wild type mice increased ERK and p38 MAPK phosphorylation was observed in the paraventricular nucleus (PVN) [24]. Another study demonstrated that repeated fasting caused activation of ERK and JNK in rat pericentral hepatocytes and hepatic macrophages [25]. Although both these two later studies did not examine the negative regulators of these MAPKs. Collectively, these observations demonstrate that fasting stress induces hepatic MKP-1 expression and selective activation and/or downregulation of p38 MAPK and JNK in different tissues as well as in distinct cell types.

Healthy cells have the ability to respond to nutrient deprivation by activating survival and stress response mechanisms/pathways and hence are resistant to a wide variety of insults such as chemotherapy, in contrast in cancer cells this type of response is attenuated [26]. Recent studies indicate that many types of cancer cells have inability to survive to fasting [27]. Although tyrosine kinase inhibitors are frequently used in the treatment of many types of cancer [28], the therapeutic benefits are not long lasting. Recent studies suggest that fasting conditions enhances the potency of the frequently used tyrosine kinase inhibitors such as erlotinib and lapatinib to inhibit MAPK signaling and cancer cell growth [29]. Our observations that p38 MAPK and JNK phosphorylation were downregulated after fasting in the liver support the notion that more research/clinical studies are required to explore the benefits of fasting in improving the potency of tyrosine kinase inhibitors in cancer treatment.

The effect of fasting on the expression of lipid regulatory genes has been documented [5,30]. Our data demonstrated that hepatic MKP-1 deficiency impairs fasting-induced changes in the levels of key transcription factors involved in the regulation of fatty acid synthesis and cholesterol metabolism. Although we did not observe differences in nuclear proteins levels of *Srebf2* between livers of MKP1-LKO and *Mkp-1^fl/fl^* mice after 24 h of fasting, the expression of its target genes including HMGCR and HMGCS were significantly reduced in MKP-LKO mice. Interestingly, when mice were refed a high carbohydrate/low fat diet, the hepatic HMGCR and HMGCS mRNA levels significantly reduced (3 h refed) in MKP1-LKO compared with *Mkp-1^fl/fl^* mice. There are many mechanisms that regulates.

*Srebf2* and its target genes. It is possible that in MKP1-LKO mice, fasting induced both synthesis and cleavage of *Srebf2*, or it may be due to decrease in the rate of degradation of *Srebf2*. mTORC1 induces nuclear *Srebf2* expression. It is conceivable that in MKP-LKO mice, mTORC1 may be fully activated leading to increased *Srebf2* levels in the fed state. Also, it is possible that MKP1-LKO has defects in bile acid synthesis which could alter cholesterol accumulation thereby affecting *Srebf2* expression. Although, *Srebf1c* expression decreased in MKP1-LKO mice after fasting, when mice were refed with high carbohydrate/low fat diet, the expression of *Srebf1c* target gene FASN were similar in *Mkp-1^fl/fl^* and MKP1-LKO mice. These observations about *Srebf1c* expression and its target gene suggest that its contribution to the metabolic effects of MKP-1 deficiency in the fasting liver is minimal.

Our results demonstrated that 24 h fast induces metabolic changes in MKP1-LKO including reduced expression of key fasting, lipid regulatory genes and transcription factors. In pathological conditions in neurons, it has been shown that high glucose levels phosphorylate MAPKs through glucose-induced oxidative stress and here we showed elimination of these conditions by fasting downregulated p38 MAPK and JNK [31].

## 6. Conclusions

Our data demonstrated that MKP-1 deficiency alters the hepatic response to fasting. These findings support the hypothesis that upregulation of MKP-1 is a physiological relevant response and might be beneficial in hepatic lipid utilization during fasting in the liver. These data suggest that MKP-1 expression is enhanced in response to changes in nutritional cues, including fasting and excess nutrients. Our data that p38 MAPK and JNK phosphorylation were downregulated after fasting in the liver support the benefits of fasting in improving the potency of tyrosine kinase inhibitors in cancer treatment. Upregulation of MKP-1 in fasting would inhibit the immune response and this could be beneficial since inflammation contributes to the pathogenesis of metabolic diseases.

## Figures and Tables

**Table 1:** The components and nutrient profile of high carbohydrate low fat diet given to mice in the study.

Ingredient	kcal./g	g/kg	kcal./kg
Casein	3.58	140	501.2
L-Cystine	4	1.8	7.2
Sucrose	4	100	400
Cornstarch	3.6	465.692	1676.4912
Dyetrose	3.8	155	589
Soybean Oil	9	40	360
t-Butylhydroquinone	0.008	0	
Cellulose	0	50	0
Mineral Mix	210,050 0.84	35	29.4
Vitamin Mix	310,025 3.87	10	38.7
Choline Bitartrate		2.5	0
		1000	3601.9912

**Table 2:** The components and nutrient profile of chow diet given to mice in the study.

Ingredient	kcal./g	g/kg	kcal./kg
Casein	3.58	200	716
L-Cystine	4	3	12
Sucrose	4	350	1400
Cornstarch	3.6	315	1134
Dyetrose	3.8	35	133
Soybean Oil	9	25	225
t-Butylhydroquinone	0	0.005	0
Lard	9	20	180
Cellulose	0	50	0
Mineral Mix #210088	1.6	10	16
Dicalcium Phosphate	0	13	0
Calcium Carbonate	0	5.5	0
Potassium Citrate H_2_O	0	16.5	0
Vitamin Mix # 300050	3.92	10	39.2
Choline Bitartrate	0	2	0
		1055.005	3855.2

## References

[1] Arroyo-Johnson C., Mincey K.D. (2016). Obesity Epidemiology Worldwide. Gastroenterol. Clin. N. Am..

[2] Andolfi C., Fisichella P.M. (2018). Epidemiology of Obesity and Associated Comorbidities. J. Laparoendosc. Adv. Surg. Tech. A.

[3] Lawan A., Bennett A.M. (2017). Mitogen-Activated Protein Kinase Regulation in Hepatic Metabolism. Trends Endocrinol. Metab..

[4] Hillgartner F.B., Salati L.M., Goodridge A.G. (1995). Physiological and molecular mechanisms involved in nutritional regulation of fatty acid synthesis. Physiol. Rev..

[5] Shimano H. (2009). SREBPs: Physiology and pathophysiology of the SREBP family. FEBS J..

[6] DeBose-Boyd R.A., Ye J. (2018). SREBPs in Lipid Metabolism, Insulin Signaling, and Beyond. Trends Biochem. Sci..

[7] Johnson B.M., DeBose-Boyd R.A. (2018). Underlying mechanisms for sterol-induced ubiquitination and ER-associated degradation of HMG CoA reductase. Semin. Cell Dev. Biol..

[8] Brown A.E., Palsgaard J., Borup R., Avery P., Gunn D.A., De Meyts P., Yeaman S.J., Walker M. (2015). p38 MAPK activation upregulates proinflammatory pathways in skeletal muscle cells from insulin-resistant type 2 diabetic patients. Am. J. Physiol.-Endocrinol. Metab..

[9] Bennett A.M., Lawan A. (2020). Improving Obesity and Insulin Resistance by Targeting Skeletal Muscle MKP-1. J. Cell. Signal..

[10] Kotzka J., Knebel B., Haas J., Kremer L., Jacob S., Hartwig S., Nitzgen U., Muller-Wieland D. (2012). Preventing phosphorylation of sterol regulatory element-binding protein 1a by MAP-kinases protects mice from fatty liver and visceral obesity. PLoS ONE.

[11] Lawan A., Shi H., Gatzke F., Bennett A.M. (2013). Diversity and specificity of the mitogen-activated protein kinase phosphatase-1 functions. Cell. Mol. Life Sci..

[12] DeBose-Boyd R.A. (2018). Significance and regulation of lipid metabolism. Semin. Cell Dev. Biol..

[13] Goldstein I., Hager G.L. (2015). Transcriptional and Chromatin Regulation during Fasting—The Genomic Era. Trends Endocrinol. Metab..

[14] Kim J.B., Sarraf P., Wright M., Yao K.M., Mueller E., Solanes G., Lowell B.B., Spiegelman B.M. (1998). Nutritional and insulin regulation of fatty acid synthetase and leptin gene expression through ADD1/SREBP1. J. Clin. Invest..

[15] Shimomura I., Shimano H., Korn B.S., Bashmakov Y., Horton J.D. (1998). Nuclear sterol regulatory element-binding proteins activate genes responsible for the entire program of unsaturated fatty acid biosynthesis in transgenic mouse liver. J. Biol. Chem..

[16] Weber L.W., Boll M., Stampfl A. (2004). Maintaining cholesterol homeostasis: Sterol regulatory element-binding proteins. World J. Gastroenterol..

[17] Pawar A., Botolin D., Mangelsdorf D.J., Jump D.B. (2003). The role of liver X receptor-alpha in the fatty acid regulation of hepatic gene expression. J. Biol. Chem..

[18] Linden A.G., Li S., Choi H.Y., Fang F., Fukasawa M., Uyeda K., Hammer R.E., Horton J.D., Engelking L.J., Liang G. (2018). Interplay between ChREBP and SREBP-1c coordinates postprandial glycolysis and lipogenesis in livers of mice. J. Lipid Res..

[19] Miao J., Haas J.T., Manthena P., Wang Y., Zhao E., Vaitheesvaran B., Kurland I.J., Biddinger S.B. (2014). Hepatic insulin receptor deficiency impairs the SREBP-2 response to feeding and statins. J. Lipid Res..

[20] Lawan A., Zhang L., Gatzke F., Min K., Jurczak M.J., Al-Mutairi M., Richter P., Camporez J.P., Couvillon A., Pesta D. (2015). Hepatic mitogen-activated protein kinase phosphatase 1 selectively regulates glucose metabolism and energy homeostasis. Mol. Cell. Biol..

[21] Raingeaud J., Whitmarsh A.J., Barrett T., Dérijard B., Davis R.J. (1996). MKK3- and MKK6-regulated gene expression is mediated by the p38 mitogen-activated protein kinase signal transduction pathway. Mol. Cell. Biol..

[22] Zhang X., Li S., Zhou Y., Su W., Ruan X., Wang B., Zheng F., Warner M., Gustafsson J., Guan Y. (2017). Ablation of cytochrome P450 omega-hydroxylase 4A14 gene attenuates hepatic steatosis and fibrosis. Proc. Natl. Acad. Sci. USA.

[23] Ajabnoor G.M., Bahijri S., Shaik N.A., Borai A., Alamoudi A.A., Al-Aama J.Y., Chrousos G.P. (2017). Ramadan fasting in Saudi Arabia is associated with altered expression of CLOCK, DUSP and IL-1alpha genes, as well as changes in cardiometabolic risk factors. PLoS ONE.

[24] Morikawa Y., Ueyama E., Senba E. (2004). Fasting-induced activation of mitogen-activated protein kinases (ERK/p38) in the mouse hypothalamus. J. Neuroendocrinol..

[25] Nishio H., Kuwabara H., Mori H., Suzuki K. (2002). Repeated fasting stress causes activation of mitogen-activated protein kinases (ERK/JNK) in rat liver. Hepatology.

[26] Lee C., Raffaghello L., Brandhorst S., Safdie F.M., Bianchi G., Martin-Montalvo A., Pistoia V., Wei M., Hwang S., Merlino A. (2012). Fasting cycles retard growth of tumors and sensitize a range of cancer cell types to chemotherapy. Sci. Transl. Med..

[27] Cheng C.W., Adams G.B., Perin L., Wei M., Zhou X., Lam B.S., Da Sacco S., Mirisola M., Quinn D.I., Dorff T.B. (2014). Prolonged fasting reduces IGF-1/PKA to promote hematopoietic-stem-cell-based regeneration and reverse immunosuppression. Cell Stem Cell.

[28] Gross S., Rahal R., Stransky N., Lengauer C., Hoeflich K.P. (2015). Targeting cancer with kinase inhibitors. J. Clin. Investig..

[29] Caffa I., Longo V.D., Nencioni A. (2015). Fasting plus tyrosine kinase inhibitors in cancer. Aging (Albany NY).

[30] Moslehi A., Hamidi-Zad Z. (2018). Role of SREBPs in Liver Diseases: A Mini-review. J. Clin. Transl. Hepatol..

[31] Purves T., Middlemas A., Agthong S., Jude E.B., Boulton A.J., Fernyhough P., Tomlinson D.R. (2001). A role for mitogen-activated protein kinases in the etiology of diabetic neuropathy. FASEB J..

